# Functional properties of metallomesogens modulated by molecular and supramolecular exotic arrangements

**DOI:** 10.3762/bjoc.5.54

**Published:** 2009-10-12

**Authors:** Alessandra Crispini, Mauro Ghedini, Daniela Pucci

**Affiliations:** 1 Centro di Eccellenza CEMIF.CAL-LASCAMM, CR-INSTM Unità della Calabria, Dipartimento di Scienze Farmaceutiche, Università della Calabria, Edificio Polifunzionale, Arcavacata di Rende (CS), 87036, Italy; 2 Centro di Eccellenza CEMIF.CAL-LASCAMM, CR-INSTM Unità della Calabria, Dipartimento di Chimica, Università della Calabria, Via P. Bucci Cubo 14C, Arcavacata di Rende (CS), 87036, Italy

**Keywords:** coordination complexes, functionality, liquid crystals, metallomesogens

## Abstract

New concepts for the synthesis of metallomesogens have been recently developed in order to use the metal centre as a scaffold for grafting different functionalities and inducing non-conventional shapes and properties in the resulting complexes. Our strategy was based on the synthesis of mesogenic coordination complexes whose molecular architectures are controlled by the modulation of different and tunable molecular motifs: the nature of the metal ion and the surrounding ligands as central unit, the number of flexible chains at the periphery, and the nature of counter-ions in ionic complexes. The appropriate choice of molecular construction motifs allows control at global architectures and induces pre-selected properties from the level of single molecule to supramolecular network, confirming that metal coordination provides a helpful tool for obtaining multifunctional soft materials.

## Introduction

Recent interest in designing novel soft and functional materials with more and more challenging requirements such as improved charge transport, luminescence, chirality and biological functions for high-tech applications has been directed towards the use of new mesomorphic systems [1–14]. Design principles based only on the shape and the symmetry of the mesogenic molecules is giving way to alternative concepts for achieving new molecular and supramolecular motifs able to give rise to dynamic functional properties and unusual topologies and families of mesophases. This goal has been reached through different strategies: the creation of hybrid molecular topologies [15–22]; the micro-segregation between incompatible units within molecules [15,23–25]; the development of self-organizing super and supramolecules able to generate complex hierarchical structures through specific inter- or intramolecular interactions [25–30]. The high level of functionality integrated into molecular-based electronic systems obtained by incorporating metal centres into selected organic structures [31] supports the design of metal-containing liquid crystals (metallomesogens) as an effective and helpful way to expand of the traditional range of technological applications of liquid crystals [32–37].

We have been involved for long time in the field of metallomesogens with the synthesis of cyclopalladated rod-like complexes starting from mesogenic azo and azoxybenzenes, confirming that mesomorphism of the organic precursors is preserved after complexation [38]. More recently, our work has addressed the design of new, higher performing complexes, whose structures, inaccessible for organic liquid crystalline systems, are able both to induce very low transition temperatures and to modulate their optical, electronic and thermal properties [34,36,38]. A multi-motif approach based on the spontaneous association of single tectons such as the metal-ligand central unit, the number of flexible chains at the periphery, the type of complementary ligands occupying the coordination sphere of the metal ion and the counter-ion in ionic systems, has been followed. In this paper selected examples of recently synthesised multifunctional metallomesogens are highlighted, all obtained from classical and unusual nitrogen ligands and different metal centres from across the periodic table, going from the most common Pd(II) to the scarcely used Pt(II) and Zinc(II), until the never used Ga(III). Indeed, with the nature of the metal centre being the leading actor in the design of new metallomesogens, the choice of unexplored metal centres is an effective route to a new generation of dynamically multifunctional soft materials, with higher performance than classical liquid crystals. Our interest in the synthesis of metallomesogens and their wide use in the field of material science, coupled with the fact that transition metal complexes have potential antitumor activity, led us to believe that a great number of metal-containing liquid crystals, already synthesized and analysed with respect to their chemical and physical properties, can constitute a huge database for the design of new biologically relevant complexes.

## Review

### Palladium(II) complexes

New mononuclear *ortho*-palladated complexes have been prepared in order to expand the applications of these systems towards new peculiar properties induced by innovative cyclometallating and ancillary ligands.

**Figure 1:**
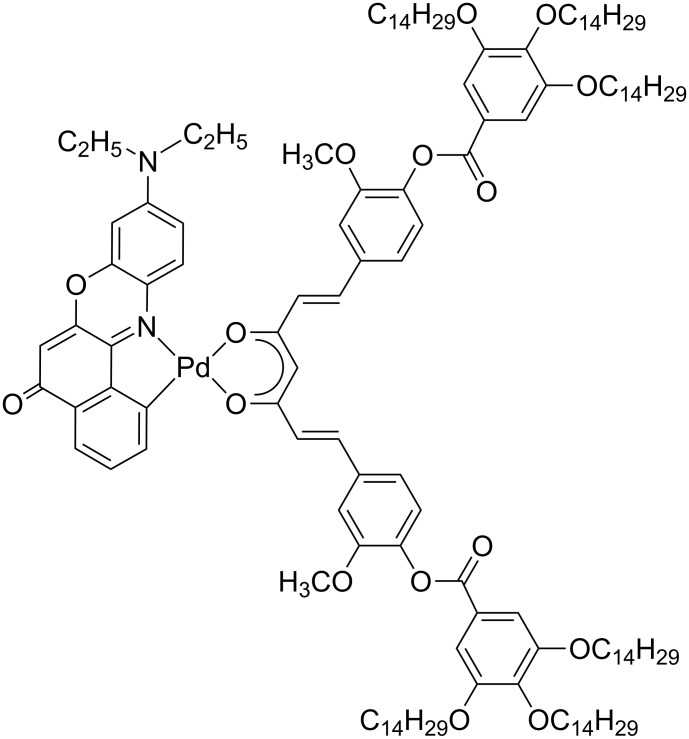
Molecular structure of NIRPAC: a Pd(II) complex based on Nile red and a curcumin derivative.

For example, an interesting red emitting mesomorphic complex has been prepared starting from the Nile red dye (9-diethylamino-5*H*-benzo[*a*]phenoxazine-5-one) as a cyclometallating ligand and from the suitably functionalized curcumin β-diketonate as a complementary O,O chelating ligand (Figure 1).

The presence of the principal ligand [39–44] introduces interesting luminescence properties into the metallic system (which is luminescent in solution), in the red region of the Vis spectrum (λ = 610 nm), with emission quantum yields in the range of 6–23%, depending on the solvent. However, the 3,4,5-trialkoxy-substituted benzoato fragment of the curcumin ligand imposes an overall hemi-disc structure to the resulting palladium derivative [45] allowing the onset, even at room temperature, of a rectangular columnar mesophase which is stable over a very large temperature range (140 °C). The emission properties observed in the solution are preserved in the liquid crystalline state, therefore the flat disk-like molecular structure organized into columns, the broad thermal stability and the luminescence in the red region of the visible spectrum make this new Pd(II) complex a very intriguing candidate for applications in OLED devices.

**Figure 2:**
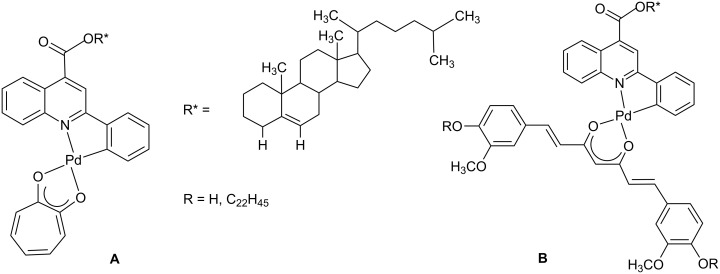
Molecular structure of Pd(II) complexes based on functionalised 2-phenylquinolines and β-diketonates.

A further class of *ortho*-palladated complexes has been obtained starting from the 2-phenylquinoline, a ligand extensively used in the synthesis of cyclometallated iridium(III) and platinum(II) derivatives [46–47] but whose reactivity towards Pd(II) centres is unexplored. This kind of ligand has been functionalised with a chiral group such as a cholesteryl ester unit, introduced as terminal substituent in 4 position, and chosen for many different purposes, including its universal affinity for cell membranes and its ability to self order into liquid crystalline state [48]. The functionalised 2-phenylquinoline ligand has firstly been cyclopalladated and then conjugated to a number of O,O chelating β-diketonate ligands, giving rise to a series of mononuclear complexes combining two chelating functional moieties in their structures (Figure 2) [49].

The coordination to the Pd(II) centre induces, in all the resulting compounds, thermotropic mesomorphism whose nature has been found to be strictly related to the ancillary ligand. Indeed a transition from a calamitic to a columnar mesophase is observed, through a calamitic/discotic cross-over point, due to the peculiar combination of two different molecular architectures. The mononuclear tropolonate derivative (A in Figure 2) shows a chiral nematic phase while the half-disc-shaped curcuminoids (B in Figure 2) self-assemble with the formation of columnar mesophases.

Moreover, the presence of biologicaly active fragments (the O,O chelating ligands) induces promising anticancer activity *in vitro* against two human prostatic cancer cell lines in all these complexes suggesting that, through the careful choice of the molecular building blocks, cyclopalladated mesogens represent multifunctional biomaterials. They bear at the same time the active principle and the membrane-compatible delivery component, and are becoming innovative tools in establishing new and effective anticancer therapies.

**Figure 3:**
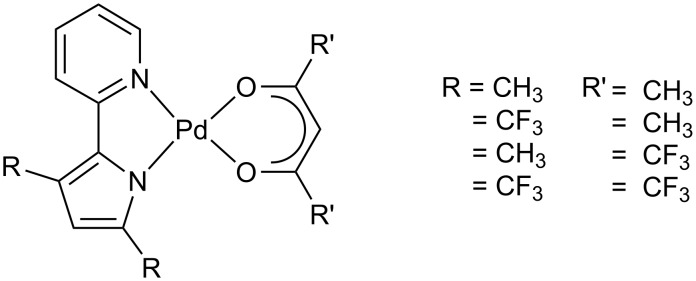
Some unusual palladiomesogens based on 3,5-disubstituted-2,2′-pyridylpyrroles and β-diketonates.

Finally, in order to investigate the unexplored cyclopalladating ability of 2,2′-pyridylpyrroles, a series of 3,5-disubstituted-2,2′-pyridylpyrroles and their mononuclear Pd(II) *ortho*-palladated derivatives (Figure 3) have been prepared [50].

The resulting complexes, containing as ancillary ligands some acetylacetonates, are “non conventional” shaped palladiomesogens. Indeed, they completely lack terminal tails, usually necessary for inducing mesomorphism. In this case the presence of fluorinated groups on the complementary ligands promotes a delicate balance between incompatible parts of the molecules, generating phase segregated structures favourable for the appearance of mesomorphism through hexagonal columnar phases. The strategy adopted, building on the modulation of incompatible simple synthons on a metallomesogenic molecule, opens new possibilities for tailoring soft materials with non-conventional structures.

### Platinum(II) and Zinc(II)

**Figure 4:**
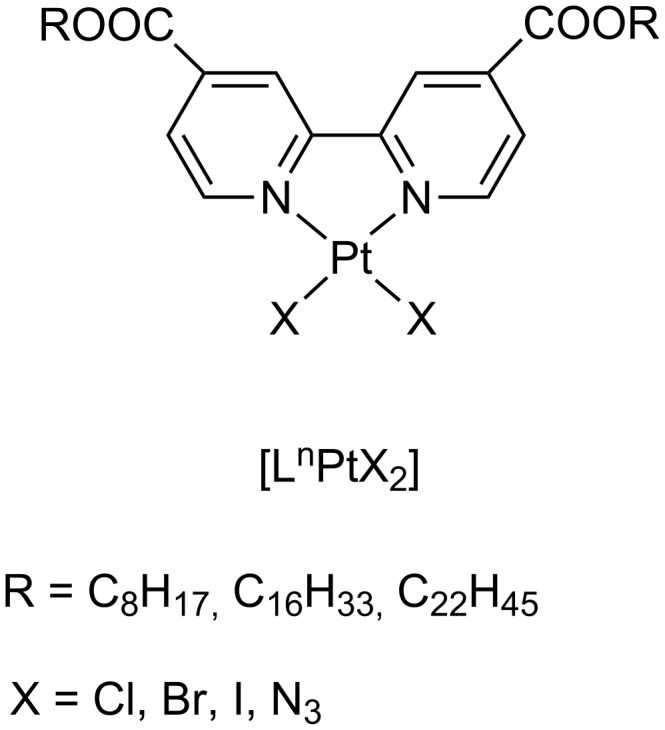
Molecular structure of Pt(II) complexes based on 4,4′-disubstituted 2,2′-bipyridines.

For the less explored Pt(II) and Zn(II) metal centres versatile 2,2′-bipyridines have been selected since they are well-known building blocks for the formation of inorganic functional nanomaterials [51]. The complexation of non-mesogenic 4,4′-disubstituted 2,2′-bipyridine ligands with Pt(II) salts confirmed the role of coordination chemistry in the metal-mediated formation of liquid crystals. Indeed the induction of a dipole moment upon coordination with an MX_2_ moiety, allowed most of the half-disc shaped complexes [L^n^PtX_2_] (Figure 4) to self-assemble into full disc shaped dimers as described by the complementary shape approach [52].

The appearance of mesomorphism is related to the length of the alkyl chains: indeed the complexes based on the 2,2′-bipyridines with short tails melted directly into an isotropic liquid, while the higher homologues produce the global rod-like shape responsible for the liquid crystalline behaviour, namely of a lamello-columnar type. Changes of the ancillary ligands have been carried out in order to use dipole coupling as a tool for molecular architecture. Mesomorphic behaviour was found to depend on the size of the X group and on the dipole moment associated with the Pt-X bond, with the sequence, for the clearing points Cl<Br<I and the azide group which promoted a lowering of both transition temperatures. Moreover, these Pt(II) complexes revealed to be photoluminescent with a good degree of tunability, depending on the π-donor capacity of the X ligands.

**Figure 5:**
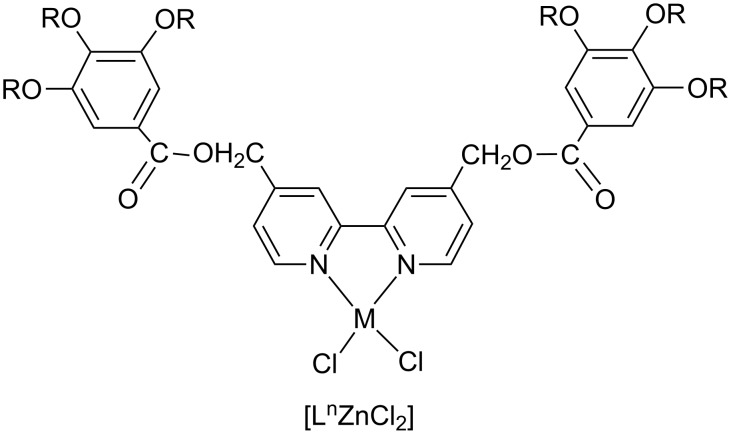
Molecular structure of Zn(II) complexes based on polycatenar 4,4′-disubstituted 2,2′-bipyridines.

Zinc(II) complexes are widely applied in OLED technology for their light emitting efficiency, high thermal and redox stability, and tunable electronic properties [53–55]. Hence the design of Zn(II) complexes showing at the same time, order, mobility, and changes in molecular organization in response to external stimuli could be a good strategy for developing new soft materials for innovative applications. In this context the same 2,2′-bipyridines have been coordinated to Zn(II) ions where the tetrahedral geometry of the Zn(II) derivatives prevented the single molecules self-assembling in dimers. Moreover, strong intermolecular contacts stabilized the crystalline state and no mesomorphic behaviour was observed below the melting point [52]. We decided to extend this work changing the substituents on the bipyridine ligands by introducing of further aromatic rings equipped with several aliphatic tails each. Hence the synthesis of a series of *cis*-dichloro hexacatenar Zn(II) complexes has been performed (Figure 5) [56].

In this case the molecular organization in the mesophase, mainly driven by intermolecular attractive interactions (hydrogen bonds, C–H–π and π–π contacts) between the large flat aromatic cores rather than dipolar or metal-metal interactions, is able to produce supramolecular columnar mesomorphism, appearing for the first time in tetrahedral Zn(II) derivatives.

These results show more and more that, by careful choice of molecular building blocks, it is possible to modulate the interactions necessary for organization of single molecules in to supramolecular architectures to give rise to the desired metallomesogenic material. Moreover, preliminary measurements of photoconductivity on these complexes doped with C_60_ to increase absorption in the visible region, have given excellent results and further experiments are still in progress.

### Gallium (III)

Since the nature of the metal centre represents an important tool for tailoring specific molecular shapes and topologies, we have selected the Ga(III) ion, never used before in the design of metallomesogens, in order to explore the possibility of inducing mesomorphism in luminescent metal complexes [57].

**Figure 6:**
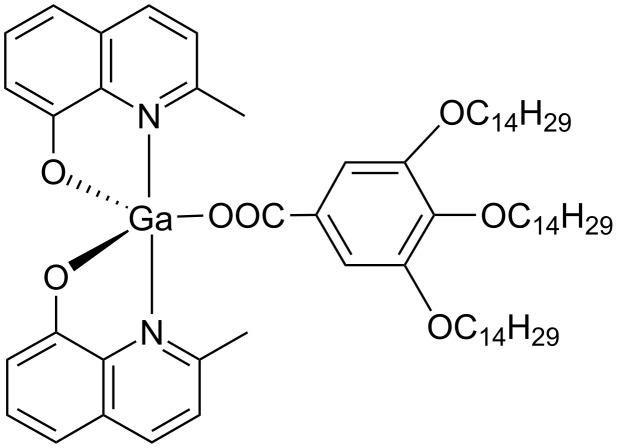
Molecular structure of a gallium(III) mesogen.

In particular, we have explored the possibility of promoting mesomorphism in pentacoordinate bisquinolinate Ga(III) coordination compounds, well-known blue emitting species, whose properties derive from solid state interactions [58–60]. Hence we have introduced the mobility of a promesogenic polycatenar group through the 3,4,5-tris(tetradecyloxy)benzoyloxy monodentate ligand in the carboxylate unit, keeping the two quinolinate groups responsible for intermolecular interactions in the crystalline organization grafted around the gallium(III) centre [61]. In this way a unconventional jellyfish shape molecule has been obtained and its molecular structure has been confirmed through single crystal X-ray diffraction (Figure 6).

Despite its unusual molecular shape, this complex shows thermotropic mesomorphism, with a lamello-columnar organization and, at the same time, a light-green emission with the typical high quantum yield of pentacordinated Ga(III) complexes (40% in solution) [62], has been detected.

### Conclusions

We have selected some examples of recent works on metallomesogens demonstrating that, moving across the periodic table, it is possible to expand the pool of metal ions able to induce mesomorphism in an appropriate framework. Hence it is possible to modulate single molecular geometries starting from the conventional square planar ion [Pd(II)], and going to the tetrahedral [Zn(II)] and pentacoordinate Ga(III) to obtain novel, non conventional structures. Moreover, by changing the nature of coordinating ligands and the kinds of substituents, further properties such as luminescence and bioactivity can be promoted at the same time, leading to smart multifunctional material. Finally, through the appropriate choice of crucial single synthons, it is possible to modulate the role of intermolecular interactions in the resulting architecture in order to create new supramolecular arrays with peculiar properties arising either from individual building blocks or from their synergy.
